# Prevalence of female genital mutilation and associated factors among daughters aged 0–14 years in sub-Saharan Africa: a multilevel analysis of recent demographic health surveys

**DOI:** 10.3389/frph.2023.1105666

**Published:** 2023-09-12

**Authors:** Asteray Assmie Ayenew, Ben W. Mol, Billie Bradford, Gedefaw Abeje

**Affiliations:** ^1^ Department of Obstetrics and Gynecology, Monash University, Clayton, VIC, Australia; ^2^ Aberdeen Centre for Women’s Health Research, Institute of Applied Health Sciences, School of Medicine, Medical Sciences and Nutrition, University of Aberdeen, Aberdeen, United Kingdom; ^3^ Department of Reproductive Health, College of Medicine and Health Sciences, School of Public Health, Bahir Dar University, Bahir Dar, Ethiopia

**Keywords:** female genital mutilation, daughter, multilevel Poisson regression, demographic health survey, sub-Saharan Africa

## Abstract

**Background:**

Female genital mutilation (FGM) is a harmful traditional practice involving the partial or total removal of external genitalia for non-medical reasons. Despite efforts to eliminate it, more than 200 million women and girls have undergone FGM, and 3 million more undergo this practice annually. Tracking the prevalence of FGM and identifying associated factors are crucial to eliminating the practice. This study aimed to determine the prevalence of FGM and associated factors among daughters aged 0–14 years.

**Methods:**

The most recent Demographic Health Survey Data (DHS) datasets from sub-Saharan African countries were used for analysis. A multilevel modified Poisson regression analysis model was applied to identify factors associated with FGM. Data management and analysis were performed using STATA-17 software, and the pooled prevalence and adjusted odds ratio (AOR) with a 95% confidence interval (CI) were reported. Statistical significance was set at *p* ≤ 0.05.

**Results:**

The study included a weighted sample of 123,362 participants. The pooled prevalence of FGM among daughters aged 0–14 years in sub-Saharan Africa was found to be 22.9% (95% CI: 16.2–29.6). The daughter's place of birth (AOR = 0.54, 95% CI: 0.48–0.62), mother's age (AOR = 1.72, 95% CI: 1.4–2.11), father's education (AOR = 0.92, 95% CI: 0.87–0.98), mother's perception about FGM (AOR = 0.42, 95% CI: 0.35–0.48), FGM as a religious requirement (AOR = 1.23, 95% CI: 1.12–1.35), mother's age at circumcision (AOR = 1.11, 95% CI: 1.01–1.23), residing in rural areas (AOR = 1.12, 95% CI: 1.05–1.19), and community literacy level (AOR = 0.90, 95% CI: 0.83–0.98) were factors associated with FGM.

**Conclusion:**

The high prevalence of FGM among daughters aged 0–14 years in sub-Saharan Africa indicates the need for intensified efforts to curb this practice. Addressing the associated factors identified in this study through targeted interventions and policy implementation is crucial to eradicate FGM and protect the rights and well-being of girls.

## Background

Female genital mutilation (FGM) refers to the deliberate removal or alteration of all or some of the female external genitalia or causing other injuries, not for medical reasons, but rather for cultural, social, or religious motives, and it does not offer any health advantages (1, 2). This harmful traditional practice has been in existence for centuries and is known by various names within different practicing communities, such as female genital cutting, circumcision, and Sunna (3).

Female genital mutilation is typically carried out on girls at a very young age, often between 7 and 8 days old up to 15 years. This is often done to prevent them from questioning or resisting the practice as older girls might do, however, it is important to note that FGM can occur at any age (4). Traditionally, all forms of FGM are performed by traditional circumcisers, commonly women, who lack medical training, and the procedures are conducted in non-sterile and unsanitary conditions, often within the confines of a girl or women's home and without any form of anaesthesia, subjecting girls to extreme pain (5). For performing FGM, local materials such as razor blades, broken glass, cow horns, scissors, knives, rocks, sharpened gouges, and other wooden materials are used (6, 7).

In the more severe forms of FGM, only a tiny opening of 2–3 mm is intentionally left, with the rest of the vulva being closed using surgical thread or thorns. Subsequently, the wound is covered with a poultice of raw egg, herbs, and salt which aid in the healing process (8). As the wound heals, a twig or similar local material may be utilized to create a small hole for urination and menstrual flow, the girl's legs are tied together often for weeks. This distressing procedure results in physical and emotional suffering for girls with long-lasting consequences on their health and well-being (9).

Female genital mutilation remains a significant public health problem that requires further investigation and collaborative efforts to eliminate (10). This practice violates fundamental human rights, including the right to life, equality, and dignity, as well as the prohibition of torture, cruel actions, and gender-based discrimination. Importantly, the consent of the child is never obtained before subjecting them to this harmful procedure (11). Despite being considered an act of violence, FGM is still prevalent in certain countries and communities (12).

The scale of this problem is staggering, with over 200 million girls and women worldwide having already undergone FGM and an additional 3 million at risk of undergoing this unacceptable act each year (13). This means that, on average, four girls continue to be subjected to this mutilation every minute (14, 15). Furthermore, various factors, such as limited resources, armed conflicts, and the impact of the COVID-19 pandemic, have further exacerbated the risk, potentially leading to even more girls falling victim to FGM (16, 17).

Female genital mutilation is predominantly practiced within certain ethnic groups in Africa, the Middle East, and some Asian and Latin American countries (18, 19). The highest concentration of this practice is found in Sub-Saharan African countries, where it thrives due to strong sociocultural forces, limited resources, and widespread illiteracy, which enable the secretive perpetration of the act and underreporting (12, 20). Despite its regional concentration, FGM has also become more globally distributed due to factors like migration and refugee movements (21, 22).

Female genital mutilation inflicts a multitude of immediate and long-term complications on girls, women, and the family (23). The procedure can lead to severe bleeding, severe pain, HIV/AIDS, tetanus, and hepatitis infection (24–27), along with shock (neurogenic, haemorrhagic, or septic) (28). Furthermore, women and girls who have undergone genital mutilation may encounter menstrual difficulties, painful and prolonged periods, nerve damage in adjacent areas, harm to the urethra, poor urinary flow, and recurrent urinary tract infections (19, 29). The adverse effects of FGM are profound and have lasting impacts on the physical, psychological, and reproductive health of affected individuals (30).

In resource-limited settings, where access to proper antenatal care and knowledge is often limited, FGM further exacerbates the high rate of complications during pregnancy and childbirth. These complications include an increased risk of prolonged labour, the need for caesarean section, haemorrhage, perineal trauma, and maternal mortality and morbidity (31–37). Infants born to mothers who have undergone FGM also face elevated risks of neonatal resuscitation, low birth weights, stillbirth, and early neonatal death (38, 39).

Female genital mutilation also inflicts adverse psychological effects on affected individuals, leading to feelings of fear, depression, and anxiety (40). Moreover, the removal of sexually sensitive tissue, such as the clitoris, impairs sexual function, affecting arousal, lubrication, orgasm, satisfaction, and overall sexual function score (41). Female genital mutilation imposes substantial financial costs on healthcare systems due to the treatment of complications arising from the practice, including hemorrhage, infection, and obstetric issues. Furthermore, FGM has wide-ranging effects on mental health, education, and employment opportunities for affected women, ultimately impacting their economic productivity and the overall economic development of their communities and countries (42). The consequences of FGM extend far beyond the immediate physical harm, highlighting the urgent need for comprehensive efforts to eradicate this harmful practice and support the well-being of affected individuals and communities (43).

The perpetuation of FGM is deeply entrenched in cultural beliefs and practices, highlighting the need for comprehensive and culturally sensitive approaches to address this harmful tradition and promote gender equality (44). Its persistence is driven by a complex interplay of sociocultural factors, including social acceptance, peer pressure, and the fear of exclusion from marriage opportunities (45, 46). In certain communities, parents may feel compelled to subject their daughters to FGM to gain respect and acceptance from their society. Circumcised girls are often considered honourable and are afforded more freedom of movement (47). Moreover, men within these communities place great emphasis on the concept of virginity and faithfulness, viewing FGM as a prerequisite for women and girls to be regarded as proper, clean, and decent (48). The sight of uncircumcised girls and women facing insults and isolation can influence some adult women to accept FGM as a means of belonging to their community (12, 47).

Over the past two decades, there has been a notable rise in efforts to eliminate female genital mutilation (16, 49). This growing interest has led to the World Health Organization (WHO), governments of numerous African countries, and human rights organizations joining forces in a collaborative endeavour to put an end to FGM (50, 51). Despite the longstanding practice of FGM spanning centuries, there is a belief that it could be eradicated within a single generation and the international community has set its sights on achieving full elimination of FGM by 2030, in line with the spirit aspiration of the Sustainable Development Goal (SDG) framework (3, 16). The collective global efforts to eliminate FGM have yielded significant progress, resulting in a noticeable decline in the likelihood of a girl being subjected to FGM compared to three decades ago (50, 51). Nevertheless, due to factors like population growth, resource constraints, and other challenges, a considerable number of girls and women continue to be at risk of FGM. Consequently, this study aims to investigate the prevalence of FGM and contributing factors among daughters aged 0–14 years in Sub-Saharan African countries. The study utilized publicly available and nationally representative datasets to gain valuable insights that could potentially aid in lowering or eliminating the practice of FGM and hoped to contribute to the ongoing efforts in safeguarding the well-being and rights of girls in the region.

## Method

### Data source

This study involved a secondary data analysis of the Demographic Health Survey (DHS) dataset from 14 Sub-Saharan African countries, namely Senegal, Mauritania, Ethiopia, Gambia, Mali, Tanzania, Togo, Benin, Burkina Faso, Chad, Ghana, Côte d'Ivoire, Nigeria, and Kenya. The DHS is a nationally representative household survey conducted in over 85 countries worldwide, with sample sizes ranging from 5,000 to 30,000 households (52, 53).

Access to the dataset was obtained through MEASURE DHS at www.measuredhs.com following a brief description of the project and online registration. Both women's and children's datasets were utilized in this analysis. The age of the daughters was calculated by subtracting their date of birth from the date of the interview, as the mothers served as the respondents. Only reproductive-age women with at least one daughter aged 0–14 years were considered for this specific study.

### Study variables

#### Primary outcome

The primary outcome of this study was female genital mutilation among daughters aged 0–14 years. To drive the outcome variable, reproductive-age women who had at least one daughter were asked if the genital area of their daughter was “cut”, or “circumcised”, or “something removed”, or “nicked with nothing removed”, or “sewn closed” and the answer was coded as No = 0, Yes = 1.

#### Independent variable

We included both individual and community-level variables. Among the individual-level variables, we included the mother's age, parent's education, parental occupation, sex of the household, marital status, religion, mothers' perception about FGM, wealth index (recoded), mother's circumcision status, information about FGM, FGM as a religious requirement, media exposure (listening Radio, reading Magazine, and watching TV), place of birth, and mother's age at circumcision. Rural residency, sub-region (East, North, West, and Central Africa), country income (2022 World Bank), and community literacy level were the community-level variables considered for this study (Appendix Table A1).

### Data management and analysis

After cleaning and recoding, data analysis was conducted using STATA-17 software (54). The data were weighted using the sampling weight after appending the extracted data from 14 Sub-Saharan African countries. The pooled prevalence of female genital mutilation among daughters aged 0–14 years with a 95% confidence interval (CI) was reported using a forest plot.

The demographic health survey data had a hierarchical structure which violates the assumption of the traditional logistic regression model, i.e., the independence of observations and equal variance assumption. Hence, reproductive-age women and daughters were nested within a single cluster, and they may share similar characteristics within the cluster. This revealed that there is a need to consider the between-cluster variability by using advanced models. Therefore, modified multilevel Poisson regression analysis was employed to identify factors significantly associated with FGM among daughters aged 0–14 years. The Intra-Cluster Correlation Coefficient (ICC), Median Odds Ratio (MOR), and Proportional Change Variance (PCV) were computed to measure the variation between clusters. Model comparison was made based on deviance [−2Log-Likelihood Ratio (LLR)] since the models were nested and a model with the lowest deviance was the best-fitted model for the data (55).

Four models were constructed for the modified multilevel Poisson regression analysis. The null model, a model without the covariates, was done to determine the extent of cluster variation in FGM among daughters. Model Ⅰ, a multilevel model adjusted with individual-level variables; model Ⅱ, a multilevel model adjusted for the community-level variables; and model Ⅲ, a multilevel model fitted with both the individual and community-level variables simultaneously. Multi-collinearity was checked using the Variance Inflation factors (VIF) by computing Pseudo- linear regression analysis and revealed that there was no multi-collinearity as all variables have VIF less than five and tolerance greater than 0.1.

### Ethical clearance

Since the study involved a secondary data analysis using publicly available data from the MEASURE DHS program, ethical approval and participant consent was not required. We prepared a concept note outlining the objective and scope of the study and formally requested access to the dataset from the MEASURE DHS through their website at https://dhsprogram.com/data/dataset_admin/index.cfm. Subsequently, we obtained permission to use the dataset for our analysis as granted by the program.

## Results

### Socio-demographic characteristics

We used the most recent (2010–2021) DHS datasets of 14 Sub-Saharan African countries and a weighted sample of 123,362 was used (Table 1).

**Table 1:** Characteristics of the included countries, survey year, and sample size.

Country	Survey year	Sample size	Sub-region	Country-income
Senegal	2019/20	6,741	West Africa	Lower middle income
Mauritania	2021	6,644	West Africa	Lower middle income
Ethiopia	2016	5,800	East Africa	Low income
Gambia	2019/20	5,215	East Africa	Lower middle income
Mali	2018	5,020	West Africa	Low income
Tanzania	2015/16	9,460	East Africa	Lower middle income
Togo	2014	5,874	West Africa	Low income
Benin	2012/13	10,586	West Africa	Lower middle income
Burkina Faso	2010	16,503	West Africa	Low income
Chad	2014/15	10,619	Central Africa	Lower middle income
Ghana	2018	8,851	West Africa	Lower middle income
Coti d’ ivoer	2011	7,732	West Africa	Lower middle income
Nigeria	2018	14,081	West Africa	Lower middle income
Kenya	2014/15	12,166	East Africa	Lower middle income

World Bank 2022.

Among the mothers of daughters included in this study, 57.8% (*n* = 71,292) fell within the age group of 20–34 years, and 59.7% (*n* = 71,376) had not received any formal education. A significant majority, comprising 67.7% (*n* = 83,472), resided in rural areas, and 76.4% (*n* = 94,295) of the daughters were born at home (Table 2).

**Table 2:** Background characteristics of study participants (*n* = 123,362).

Variables	Weighted frequency	Unweighted frequency
	*N* (%)	*N* (%)
Daughter's place of birth		
Health facility	29,066 (23.56)	28,740 (22.97)
Home	94,295 (76.44)	96,386 (77)
Mother's age years		
15–19	2,700 (2.19)	2,703 (2.16)
20–34	71,292 (57.79)	69,691 (57.11)
35–49	49,368 (40.02)	50,966 (40.73)
Mother's education		
No education	71,376 (57.86)	75,028 (59.96)
Primary education	32,405 (26.27)	31,379 (25.08)
Secondary and above	19,578 (15.87)	18,719 (14.96)
Father's education		
No education	64,530 (55.23)	68,172 (57.42)
Primary education	26,045 (22.29)	25,403 (21.4)
Secondary and above	26,267 (22.48)	25,151 (21.18)
Fathers occupation		
Working	112,077 (4.2)	113,799 (95.8)
Not working	4,918 (95.8)	5,027 (4.23)
Marital status of the mother		
Single	2,417 (1.96)	2,357 (1.88)
Married	115,103 (93.31)	2,357 (93.25)
Others	5,840 (4.73)	6,090 (4.87)
Wealth index		
Poor	52,918 (42.9)	57,571 (46.01)
Middle	25,066 (20.32)	25,137 (20.09)
Rich	45,378 (36.78)	42,418 (33.90)
Sex of household		
Male	102,113 (82.78)	103,261 (82.53)
Female	21,248 (17.22)	21,865 (17.47)
Mother's occupation		
Working	13,709 (83.27)	13,883 (84.98)
Not working	2,755 (16.73)	2,454 (15.02)
FGM required by religion		
Yes	85,425 (69.25)	84,956 (67.9)
No	85,425 (25.42)	84,956 (26.75)
Not sure	6,580 (5.33)	6,705 (5.36)
Mothers’ perception about FGM stopped		
Stopped	31,190 (25.3)	32,772 (26.23)
Continued	83,595 (67.85)	83,671 (66.96)
Not sure	8,426 (6.84)	8,514 (6.81)
Respondents circumcision status		
Yes	63,677 (51.62)	65,637 (52.5)
No	59,679 (48.38)	59,487 (47.5)
Mother's age at FGM		
During Infancy	43,553 (50.72)	44,378 (50.19)
After infancy	42,313 (49.3)	44,045 (49.8)
Ever heard of FGM		
Yes	121,190 (98.24)	122,757 (98.11)
No	2,169 (1.76)	2,367 (1.89)
Listening radio		
Yes	73,295 (40.56)	72,725 (58.14)
No	50,015 (59.44)	52,358 (41.86)
Reading magazine		
Yes	25,374 (20.6)	24,583 (19.66)
No	97,908.925 (79.42)	100,470 (80.3)
Watching TV		
No	49,712 (40.4)	77,013 (61.3)
Yes	25,374 73,488 (59.7)	47,954 (38.4)
Media exposure		
Exposed	82,707.792 (67.2)	82,010 (65.68)
Non-exposed	40,387.65 (32.8)	42,860 (34.32)
Community literacy		
Low	82,619 (67)	85,754 (86.6)
Medium	10,550 (8.56)	10,312 (8.25)
High	30,124 (24.4)	28,999 (23.2)
Residency		
Urban	39,888 (32.33)	38,281 (30.59)
Rural	83,472 (67.67)	86,845 (69.41)
Country income		
Low income	54,589 (44.25)	54,236 (43.35)
Lower middle income	68,773 (55.75)	70,890 (56.65)
Sub-region		
Eastern Africa	31,788 (25.77)	32,641 (26.09)
Central Africa	10,516 (8.5)	10,619 (8.49)
West Africa	81,057 (65.7)	81,866 (65.4)

### The pooled prevalence of female genital mutilation among daughters aged 0–14 years in SSA

**Figure 1:**
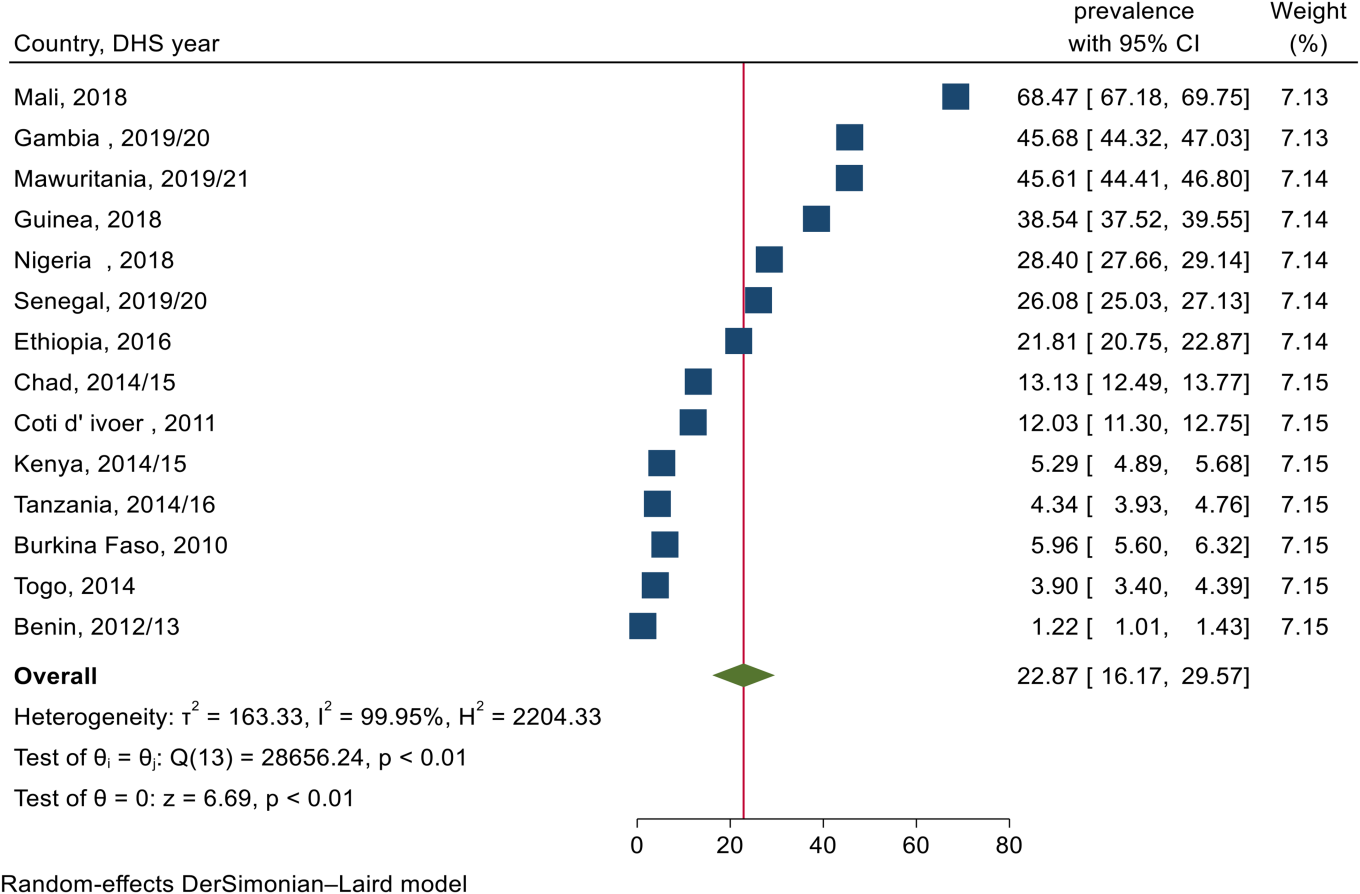
Forest plot for the pooled prevalence of female genital mutilation among daughters aged 0–14 years old in SSA.

The pooled prevalence of female genital mutilation among daughters aged 0–14 years in sub–Saharan Africa was 22.9% (95% CI: 16.2–29.6) ranging from 1.2% (95% CI: 1.0–1.4) to 68.5% (95% CI: 67.2–69.8) in Benin and Mali respectively (Figure 1).

### Random effect analysis and model fitness comparison

The random effect model's assessment was conducted using ICC, PCV, and MOR. The ICC value in the null model was 0.71%, indicating that approximately 71% of the total variation in FGM was attributable to differences between clusters, with the remaining 29% attributed to individual-level variability in FGM among daughters. Additionally, the MOR value was 10.57, suggesting significant variation in FGM between clusters. Furthermore, the Proportional change in Variance (PCV) was found to be highest in the final model, indicating that both individual and community-level variables accounted for 67% of the variation in FGM. Model III emerged as the best-fit model, boasting the lowest deviance among all the models assessed (Table 3).

**Table 3:** Random effect analysis and model comparison in the assessment of factors associated with FGM among daughters aged 0–14 years in SSA.

Parameters	Null	Model I	Model II	Model III
ICC	0.71			
MOR	10.57	4.417	8.63	3.91
PCV	Ref	0.60	0.14	0.67
Model comparison				
Log likelihood ratio (−2LL)	49,414.9	38,890.4	49,050.1	38,760

ICC, intra class correlation coefficient; MOR, median odds ratio; PCV, proportional change variance.

### Factors associated with female genital mutilation among daughters 0–14 years old in sub-Saharan Africa

We considered model Ⅲ for determining factors associated with FGM among daughters of reproductive-age women, as it had the lowest deviance. Daughters born to mothers aged between 20 and 34 and 35 and 49 years old had a 48% (adjusted odds ratio (AOR) = 1.48, 95% confidence interval (CI): 1.25–1.76), and a 72% (AOR = 1.72, 95% CI: 1.4–2.11) higher probability of experiencing FGM respectively compared with daughters whose mother were between the age of 15 and 19 years old. Considering the daughter's Place of birth, those born at a health facility had a lower chance of being circumcised (AOR = 0.54 = 95% CI: 0.48–0.62) compared to those born at home.

Daughters whose fathers completed secondary and above educational level had an 8% (AOR = 0.92, 95% CI: 0.87–0.98) lower chance of having FGM compared to daughters whose fathers had no formal education. Additionally, daughters born from mothers who perceived FGM should be stopped or were unsure had a 58% (AOR = 0.42, 95% CI: 0.35–0.48) and a 25% (AOR = 0.75, 95% CI: 0.68–0.83) lower chance of being circumcised than daughters whose mothers perceived FGM to be continued respectively.

Concerning female genital mutilation being perceived as a religious requirement, daughters whose mothers believed that FGM should be practiced as a religious requirement or were unsure had a 23% (AOR = 1.23, 95% CI: 1.12–1.35) and a 10% (AOR = 1.10, 95% CI: 1.01–1.19) higher probability of being subjected to the practice compared to daughters from mothers who did not indicate that FGM should be practiced as a religious requirement.

Daughters whose mothers underwent circumcision during infancy had an 11% (AOR = 1.11, 95% CI: 1.01–1.23) higher chance of being circumcised compared to daughters whose mothers were circumcised after infancy. Furthermore, residing in a rural area was associated with a 12% higher likelihood of circumcision among daughters (AOR = 1.12, 95% CI: 1.05–1.19), compared to those living in urban areas. Additionally, daughters living in communities with higher literacy had a 10% lower chance of being circumcised, 10% (AOR = 0.90, 95% CI: 0.83–0.98) compared to daughters residing in communities with lower literacy levels (Table 4).

**Table 4:** Multivariable multilevel modified poisson regression analysis of female genital mutilation among daughters of reproductive age women aged 0–14 in sub-Saharan African countries.

Variables	Daughter's circumcision		Null model^n^	Modell II^I^	Modell II^c^	Modell III^f^
	Yes *N* (%)	No *N* (%)	AOR (95% CI)	AOR (95% CI)	AOR (95% CI)	AOR (95% CI)
Wealth index						
Poor	13,247 (10.6)	44,373 (35.4)		1		1
Middle	4,854 (3.9)	20,326 (16.2)		0.91 (0.96–1.03)		1.00 (0.97–1.05)
Rich	6,190 (4.9)	36,302 (29)		0.90 (0.91–1.04)		1.00 (0.96–1.14)
Mother's age in 5 years group						
15–19	334 (0.27)	2,374 (1.9)		1		
20–34	12,767 (10.2)	58,782 (46.9)		1.51 (1.24–1.75)***		1.48 (1.25–1.76)***
35–49	11,190 (8.9)	39,845 (31.8)		1.66 (1.39–2.0)***		1.7 2 (1.4–2.11)***
Educational status of the mother						
Uneducated	17,992 (14.4)	57,179 (45.6)		1		
Primary	3,770 (3)	27,625 (22)		0.96 (0.88–1.0)		0.98 (0.93–1.03)
Secondary+	2,529 (2)	16,197 (13)		0.91 (0.85–0.97)*		1.04 (0.95–1.15)
Educational status of the father						
No education	17,640 (14.9)	50,666 (42.6)		1		
Primary education	2,373 (2)	23,060 (19.4)		0.93 (0.88–1.0)*		0.94 (0.78–1.04)
Secondary education and above	3,336 (2.8)	21,816 (18.4)		0.90 (0.86–0.97)***		0.92 (0.87–0.98)***
Sex of household						
Male	20,284 (19.2)	83,135 (66.4)		1		
Female	4,007 (14.3)	17,866 (14.3)		1.02 (0.99–1.06)		1.03 (0.99–1.07)
Mothers circumcision status						
Yes	22,278 (17.8)	43,492 (34.7)		2.71 (1.8–3.96)***		2.7 (1.86–4.0)***
No	2,013 (1.6)	57,507 (45.9)		1		
Mothers’ perception of FGM						
Continued	17,654 (15)	15,142 (12.1)		1		1
Stopped	5,019 (4)	78,775 (63)		0.40 (0.35–0.48)***		0.75 (0.68–0.83)***
Not sure	1,599 (1.28)	6,934 (5.6)		0.70 (0.67–0.83)***		0.42 (0.35–0.48)***
Required by religion						
No	7,541 (6.02)	77,415 (61.8)		1		1
Yes	151,180 (12.1)	18,451 (14.7)		1.21 (1.12–1.35)***		1.23 (1.12–1.35)***
Don't know	1,570 (1.25)	5,135 (4.1)		1.10 (1.01–1.19)**		1.10 (1.01–1.19)**
Husband/partner's occupation						
Working	21,559 (18.1)	92,403 (77.7)		1		0.99 (0.95–1.04)
Not working	1,818 (1.5)	3,211 (2.7)		0.99 (0.95–1.04)		1
Mother's occupation						
Working	15,616 (12.8)	71,977 (58.8)		1.00 (1.0–1.1)**		1.03 (0.99–1.07)
Not working	7,830 (6.4)	26,905 (23)		1		1
Media exposure						
Yes	14,921 (11.9)	67,195 (53.7)		0.99 (0.95–1.04)		1.10 (0.96–1.05)
No	9,339 (7.5)	33,581 (26.9)		1		1
Place of birth						
Health facility	2,859 (2.3)	25,948 (20.7)		0.50 (0.54–0.62)***		0.54 (0.48–0.62)***
Home	21,432 (17.1)	75,061 (60)		1		
Mothers’ age at circumcision						
Not infancy	10,009 (11.3)	34,107 (38.5)		1		
During infancy	13,935 (15.7)	30,505 (34.5)		1.10 (1.09–1.1)**		1.11 (1.01–1.23)***
Heard FGM						
Yes	24,213 (19.3)	2,289 (1.8)		1		1
No	78 (0.1)	2,289 (1.8)		1.40 (0.98–1.86)		1.36 (0.99–1.89)
Residency						
Urban	6,223 (5)	32,098 (25.6)			1	
Rural	18,068 (14.4)	68,903 (55)			1.20 (1.1–1.3)***	1.12 (1.05–1.19)***
Country-income						
Low income					1	
Lower middle income					1.70 (0.82–3.7)	1.24 (0.74–2.1)
Sub-region						
East Africa	4,331 (3.5)	28,310 (22.6)			1	
Central Africa	1,394 (1.1)	1,394 (7.4)			4.00 (1.6–10.2)***	1.82 (0.8–4.13)
West Africa	18,566 (14.8)	63,466 (50.7)			3.6 (2.9–4.67)***	1.75 (0.69–4.3)
Community literacy						
Low	20,072 (16%)	65,825 (52.6)			1	
Medium	1,819 (1.45%)	8,502 (6.8)			0.80 (0.78–0.9)***	0.92 (0.77–1.10)
High	2,391 (1.91)	26,622 (21.3)			0.70 (0.6–0.7)***	0.90 (0.83–0.98)**

NB: 1, refence value; CI, confidence interval; AOR, adjusted odds ratio; ICC, intra cluster correlation; PCV, percentage change variation; ^n^, null model; ^I^, individual level variable model; ^c^, community level variable model; ^f^, full mode.

* Significant at *p*-value 0.05.

** Significant at *p*-value 0.01.

*** Significant at *p*-value 0.001.

## Discussion

The pooled prevalence of female genital mutilation among daughters aged 0–14 in sub-Saharan Africa was found to be 22.9%. This figure is higher than the reported global prevalence of FGM among girls in 25 countries at 14.7% from a recent systematic review and meta-analysis (56). It is also higher than the prevalence of 13% reported from a mixed-effect multilevel analysis of demographic health surveys, as well as the prevalence of 16.3% reported in Europe (57). However, it is lower than the prevalence of 35% reported in a study conducted on female adolescents aged 13–19 years in FGM-endemic areas (58). The possible reason might be the study conducted specifically focused on areas identified as endemic for the practice of FGM. Additionally, the discrepancy in prevalence may be influenced by the difference in the study period, as this was conducted more recently.

Daughters aged 0–14 years, whose mothers were between the age of 20–34 and 35–49 years, were found to have a higher likelihood of being circumcised compared to girls whose mothers were between 15 and 19 years old. This finding is supported by various studies, including one that examined the risk of female genital mutilation in daughters (59), a hierarchical analysis investigating factors associated with a daughter's circumcision (60), as well as studies conducted in Chad, Kenya, and Burkina Faso (5, 61–63). A possible explanation for this trend could be that younger mothers might be more educated and empowered, making them more resistant to sociocultural influences and harmful traditional practices (64).

This study also found that female genital mutilation was more prevalent among daughters whose mothers had undergone circumcision during infancy compared to those whose mothers were circumcised after they grew up. This finding is supported by another study which demonstrated that women who had never experienced FGM were more likely to have their daughters circumcised (5). A possible explanation for this observation is that mothers who were circumcised at a very early age or never experienced circumcision themselves may lack understanding about the practice and its potential complications, thus potentially allowing their daughters to undergo the procedure. Conversely, women who had experienced FGM at a younger age may be more inclined to condemn the practice due to the adverse effects they have personally experienced on their health (65, 66). Further research is recommended to gain further insights into the relationship between mothers' age at circumcision and their daughters' circumcision status.

Daughters whose birth took place at a health facility were found to have a lower likelihood of experiencing genital mutilation compared to daughters born at home. This finding is consistent with a study that conducted hierarchical analysis on factors associated with a daughter's FGM (60) and as well as studies conducted in Africa (67, 68). One possible reason for this association could be that giving birth at a health facility is linked to better access to information, education, and counselling regarding the sexual and reproductive health of both the mother and daughters. It also provides postnatal care and immunization, which may contribute to reducing the prevalence of FGM among daughters. Furthermore, delivering at a health facility reduces daughters' exposure to traditional birth attendants, as over 90% of circumcision procedures are typically conducted by older women or traditional birth attendants (69, 70).

Daughters residing in rural areas were found to have a higher likelihood of experiencing genital mutilation compared to those living in urban areas. This finding was supported by several studies conducted in Sub-Saharan Africa (71, 72), a hierarchical analysis investigating factors associated with a daughter's circumcision (60), systematic reviews in Europe (73, 74), studies conducted in other regions of Africa (75), and a study in the eastern parts of Africa (76). There are potential explanations for this observation. Firstly, rural residents often have lower levels of education, which may result in limited access to information, counselling, and knowledge about harmful traditional practices, including female genital mutilation (77, 78). Secondly, within rural communities, there may be a strong commitment to preserving sociocultural traditions, leading to a reluctance to abandon practices like FGM (20, 79, 80). To address this issue, it is crucial to focus on providing education about the consequences of FGM specifically in rural areas.

Daughters aged 0–14 years, whose fathers completed secondary education and above, were found to have a reduced likelihood of undergoing FGM compared to those whose fathers had no formal education. This finding was consistently supported by various studies conducted in Sub-Saharan Africa (71), a study exploring factors associated with a daughter's circumcision (60), research by Andro et al. (79), data from the United Nations Children's Fund (81), as well as research in Iran (59, 81) and Egypt (82, 83). The possible explanation for this association lies in the fact that educated fathers are better equipped to mitigate the social pressure exerted by family members, ultimately reducing the likelihood of their daughters undergoing circumcision (84). With a higher level of education, fathers may possess a greater understanding of the adverse consequences of female genital mutilation and be more inclined to protect their daughters from this harmful traditional practice. Thus, promoting education among fathers could play a pivotal role in the collective efforts to eliminate FGM and safeguard the health and well-being of girls in these communities (85).

Daughters whose mothers perceived female genital mutilation to be continued had a higher likelihood of undergoing circumcision compared to their counterparts. This finding aligns with a study on daughter circumcision (59), a study conducted in Senegal (84), and the UNICEF report (63). The possible reason for this association lies in the fact that mothers' perception plays a crucial role in determining their intention to allow their daughters to undergo circumcision. Mothers who hold a favourable view of the continuation of FGM are more likely to permit their daughters to be circumcised (59). Furthermore, mothers may fear facing social sanctions or blame if they deviate from the prevalent practice of female genital mutilation (86, 87).

Daughters whose mothers believed that female genital mutilation must be practiced as a religious requirement had a higher likelihood of undergoing circumcision. This finding was consistent with studies conducted in Africa (60), Sub-Saharan Africa (71), and the World Health Organization report (88). The association could be explained as a culturally specific interpretation of religious identity, wherein the practice may be influenced by individual interpretations of religious doctrine (89). Additionally, the practice of FGM may be perceived as a means to control women's and girls' sexuality by suppressing their sexual desire, while religion, often emphasizing purity and decency, could inadvertently promote the continuation of this practice (90).

Daughters residing in communities with a higher literacy level were found to have a reduced likelihood of experiencing genital mutilation compared to those living in communities with a lower literacy level. This finding is supported by studies conducted in Africa (5), the World Health Organization report (91), and the UNICEF report (63). Literacy is recognized as an essential tool for fostering positive attitude changes (92). In communities with higher literacy levels, individuals are better equipped to understand health information, including the consequences of FGM. This enhanced understanding empowers women and parents to challenge harmful sociocultural norms and practices, such as subjecting daughters to FGM, even in the face of societal pressure to uphold the practice (93). Promoting literacy in communities can serve as a powerful means of promoting positive change and combating harmful practices like FGM. By fostering a better-informed population, efforts to eliminate FGM can be strengthened, leading to improved health outcomes and enhanced gender equality.

### Strengths and limitations of the study

The strength of this study is using the Demographic and Health Surveys (DHS) dataset, a nationally representative household survey with a large sample size. Furthermore, the DHS dataset offers high response rates, rigorous interviewer training, standardized data collection procedures across countries, and consistent content over time, enhancing comparability across populations both cross-sectionally and longitudinally. Employing multilevel analysis accounted for the hierarchical nature of the data, ensuring reliable estimates. However, the study also has limitations. The cross-sectional design may limit the ability to establish causality between variables, as it captures data at a single point in time. Additionally, the study's reliance on secondary data introduces potential limitations, as the data's original purpose may not align perfectly with the study's specific objectives. Despite these drawbacks, the study provides valuable insights into the prevalence and associated factors of female genital mutilation among girls, contributing to the body of knowledge addressing this critical public health issue.

## Conclusion

The prevalence of female genital mutilation among daughters aged 0–14 in sub-Saharan Africa remains high. This study has identified community and individual-level factors associated with FGM, highlighting the urgency of developing a systematic and coordinated strategy and policy to eliminate this harmful practice within one generation.

Disrupting the intergenerational trauma caused by FGM calls for targeted efforts in raising awareness and transforming social norms, religious perceptions, and attitudes. By doing so, we can pave the way toward eradicating FGM. Public health interventions must be designed to address specific risks, including daughters from older mothers, rural residents, circumcised mothers, and those living in communities with low literacy levels. Providing access to information, education, and counselling about FGM for both boys and girls is essential in fostering a broader understanding of the harmful consequences associated with the practice. Furthermore, involving men in the prevention strategy, engaging in the conversation, and encouraging their active support can have a significant impact on the success of initiatives aimed at eliminating FGM.

## Data Availability

The original contributions presented in the study are included in the article/Supplementary Material, further inquiries can be directed to the corresponding author.

## Appendix

**Table A1:** Table showing independent variables and their description/categorization.

Variables	Description
Individual-level variables	
Age of the mother	The current age of the mother was recorded into three categories (“15–19”, “20–34”, and “35–49”)
Wealth index	The dataset contained a wealth index that was created using principal component analysis and coded as “Poorest”, “Poorer”, “Middle”, “Richer”, and “Richest” in the EDHS dataset. For our study, we recorded this into the three categories “Poor” (which includes Poorest and Poorer), “Middle”, and “Rich” (which includes the Richest and Richer categories)
Working status of the parents	For both the mother and father, working status was recorded and categorized as “working” and “not working”
Daughter's Place of birth	This variable was categorized into two: “health facility” and “home”
Educational status of the parents	This is the minimum educational level that a mother and a father achieved and recorded into three groups as “no formal education”, “primary education”, and “secondary education and above”
Marital status	This was the current marital status of the mother and recorded into three categories as “Single” (including never in union), “married” (included married and living with partner), and “others” (included divorced and no longer living together with their partner)
Sex of household	The variable sex of the household was coded as “man” and “women” without change
Ever heard of FGM	The variable ever heard of FGM was coded as “yes” and “no”, used without change
Female circumcision required by religion	This variable was recoded as “yes”, “no”, and “not sure” (which includes “no religion” and “I don't know”)
Mother's perception about FGM (Female circumcision: continue or be stopped)	This variable was recoded as “continued”, “stopped”, and “not sure” (which includes “it depends” and “I don't know”)
Mother circumcision status	The variable of the mother's circumcision status was coded as “yes” and “no”, used without change
Media exposure	Media exposure was recorded after generating the sum of three media (listening to radio, watching TV, and reading magazines) per week for each, and then “yes” was given for those who had at least one media exposure, and “no” was given for those who had no exposure
Mothers age at circumcision	The variable age at circumcision was recorded as “during infancy” which includes those who had circumcision before their 1 year of birth, and “not infancy” for those who had FGM after 1 year of their childhood life
Community-level literacy	
Community literacy	The variable community literacy was recorded as “low” (including cannot read at all), “medium” (including reading only part of a sentence), and “high” (including being able to read the whole sentence)
Types of place of residence	The variable place of residence coded as “rural” and “urban” without change
Country income	The variable Country income was recorded as “low income” and “lower middle-income” according to the world bank report of 2022
Sub-region	Sub-region was recoded as five regions “Eastern” “southern” “central” “West” “North” as to UN 2022 report
